# Antibacterial Activity of Guttation Droplets from *Penicillium pimiteouiense* and *Penicillium menonorum* Against Clinically Relevant Bacterial Pathogens

**DOI:** 10.3390/jof12040262

**Published:** 2026-04-03

**Authors:** Carlos Eduardo Barajas-Saucedo, Mariana Torres-Cruz, Juan Carlos Sánchez-Rangel, Abraham Vidal-Limon, Juana María Jiménez-Vargas

**Affiliations:** 1 Facultad de Ciencias Químicas, Universidad de Colima, Colima 28400, Mexico; carlos_barajas24@ucol.mx; 2 Facultad de Ciencias Biológicas y Agropecuarias, Universidad de Colima, Colima 28930, Mexico; mariancruzt0@gmail.com (M.T.-C.); jsanchez4@ucol.mx (J.C.S.-R.); 3 Red de Estudios Moleculares Avanzados, Instituto de Ecología A.C. (INECOL A.C.), Carretera Antigua a Coatepec 351, Xalapa 91073, Mexico; abraham.vidal@inecol.mx; 4 Laboratorio Nacional de Plantas Medicinales (LANPLAM), Secretaría de Ciencia, Humanidades, Tecnología e Innovación (SECIHTI), Carretera Antigua a Coatepec 351, Xalapa 91073, Mexico; 5 Secretaría de Ciencia, Humanidades, Tecnología e Innovación (SECIHTI), Av. Insurgentes Sur 1582, Col. Crédito Constructor, Bénito Juárez, Mexico 03940, Mexico

**Keywords:** antimicrobial discovery, antibacterial natural products, antimicrobial resistance, fungal guttation, fungal secondary metabolites, *Penicillium* metabolites

## Abstract

Antimicrobial resistance (AMR) represents a major global health challenge, driving the search for novel antimicrobial compounds from natural sources. Filamentous fungi are prolific producers of bioactive metabolites, yet the biological potential of fungal guttation droplets remains relatively underexplored. In this study, guttation droplets produced by *Penicillium pimiteouiense* and *Penicillium menonorum*, isolated from rhizospheric soils of *Opuntia* spp. in Colima, Mexico, were evaluated for antibacterial activity against clinically relevant pathogens. Fungal isolates were identified through macromorphological characterization and sequence analysis of ITS and β-tubulin genes. Antibacterial activity of the guttation droplets was evaluated using agar well diffusion and microbroth dilution assays to determine inhibition zones, minimum inhibitory volume (MIV), and minimum bactericidal volume (MBV). The exudates exhibited measurable activity against several Gram-negative and Gram-positive bacteria, including *Escherichia coli*, *Salmonella enterica*, *Klebsiella pneumoniae*, *Serratia marcescens*, and *Staphylococcus aureus*. Guttation droplets from *P. pimiteouiense* showed the highest inhibition, with zones up to 24.4 mm against *S. enterica*, and activity comparable to gentamicin. MBV/MIV ratios indicated bactericidal activity against selected pathogens, including *E. coli*, *K. pneumoniae*, and *S. aureus*. These findings demonstrate that fungal guttation droplets represent a promising and underexplored source of antibacterial compounds and support their potential for antimicrobial discovery.

## 1. Introduction

Antimicrobial resistance (AMR) is a natural process driven by genetic changes in various pathogens over time. It represents one of the most critical global threats to public health in the twenty-first century [1]. However, human activities significantly accelerate this process, primarily through the overuse or misuse of antibiotics for treatment, prevention, or control of infections across the agricultural, animal, and human health sectors. The escalating threat of multidrug-resistant (MDR) bacteria has become a critical, global public health challenge. In 2021, AMR bacteria were estimated to be responsible for 4.71 million deaths, and this number is projected to reach 10 million by 2050 [2,3]. In response to this escalating crisis, the World Health Organization (WHO) updated its Bacterial Priority Pathogens List (BPPL) in 2024, identifying 24 bacterial species of major clinical concern due to their high levels of resistance and limited treatment alternatives. This list includes critical and high-priority pathogens such as *Klebsiella pneumoniae*, *Escherichia coli*, *Acinetobacter baumannii*, *Mycobacterium tuberculosis*, *Salmonella enterica* serotype Typhi, *Shigella* spp., *Enterococcus faecium*, non-typhoidal *Salmonella*, *Enterobacter* spp., *Neisseria gonorrhoeae*, and *Staphylococcus aureus*, among others [4].

In this critical context, filamentous fungi remain among the most prolific and chemically diverse sources of bioactive compounds with antimicrobial potential [5,6]. Increasing attention has therefore been directed toward unexplored fungal metabolic pathways and alternative secretion mechanisms that may contribute to the discovery of new antimicrobial scaffolds, such as the secretion of secondary metabolites via guttation, which in certain fungi constitutes a natural and efficient pathway for producing molecules with diverse biological activities [7,8,9,10,11]. In particular, guttation fungal droplets (enzymes, ammonia, oxalic acid, and water, alongside secondary metabolites) that serve multiple roles, including protection against UV light, elimination of toxic molecules, water conservation, and facilitation of communication and signaling [5,12].

The evaluation of the biological activity of fungal droplets has become increasingly important in recent years. For example, exudates from *Penicillium expansum* and *Trichoderma atroviride* have been shown to exhibit cytotoxic activity associated with the enzyme acetylxylan esterase [13]. Furthermore, antibacterial activity has been observed in the exudates of several species, including *Pseudoxylaria* sp. (pseudoxylalemycins and epoxy-cytochalasins) [14], *Penicillium restrictum *(ω-hydroxyemodin, polyhydroxyanthraquinone) [15], *Penicillium citreonigrum* (sclerotiorin and sclerotioramine) [16], *Penicillium brevicompactum* (defensin) [17]. The compounds identified as responsible for this activity include polyhydroxyanthraquinones, dextrusins, and defensins, respectively [14,16,17,18]. Additionally, pentaibol, the Trichogin GA IV produced by *Trichoderma longibrachiatum*, which is recognized as one of the most potent antibacterial compounds, has also been isolated from fungal exudates [19,20].

Our aim is to investigate the antibacterial properties of guttation droplets produced by two fungal species found in the rhizosphere microenvironment of *Opuntia ficus-indica* (prickly pear). This guttation exhibits significant activity against a panel of clinically relevant Gram-positive and Gram-negative bacterial pathogens. Our findings reveal that fungal guttation droplets function as a biologically active extracellular matrix with significant antibacterial activity, underscoring their potential importance as a promising source of natural antimicrobial products. Consequently, these results are anticipated to lead to the identification of novel bioactive components that can help address the global challenge of multidrug resistance.

## 2. Materials and Methods

### 2.1. Bacterial Strains, Medium and Reagents

For the antibacterial evaluation, the following reference strains were utilized: *Escherichia coli* ATCC 25922, *Escherichia coli* ATCC 35218, *Salmonella enterica* serotype Typhimurium ATCC 14028, *Staphylococcus aureus* ATCC 25923, and *Enterococcus faecalis* ATCC 29212. Additionally, clinical isolates of *Klebsiella pneumoniae*, *Serratia marcescens*, *Proteus mirabilis*, and *Citrobacter freundii* were also included in the assays. These were obtained from the Clinical Laboratory of the Faculty of Chemical Sciences at the University of Colima (Colima, Mexico). Clinical isolates used in this study were obtained from routine diagnostic laboratory procedures and were fully anonymized. Ethical review approval was waived for this study, as no patient-identifiable information was accessed, in accordance with institutional guidelines and the declaration of Helsinki.

The culture media and reagents used in this study were: Modified Potato Dextrose Agar (MCD LAB, Tlalnepantla de Baz, Mexico), Yeast Extract (BD BBL, Franklin Lakes, NJ, USA), chloramphenicol (QuimiNet, Mexico, Mexico), Mueller-Hinton Broth (BMH), and Mueller-Hinton Agar (AMH) (BD DIFCO^®^, Mexico, Mexico), 0.85% sterile saline (PISA, Mexico, Mexico), gentamicin (AMSA, Mexico, Mexico), Trypticase in Soy Agar (TSA), and Tryptic Soy Broth (TSB) (BD DIFCO, Mexico), MacConkey Agar (MCA; BD BIOXON, Mexico, Mexico), Mannitol Salt Agar (MSA; BD BIOXON, Mexico). For the well-diffusion positive control, gentamicin discs (10 µg; BD, Franklin Lakes, NJ, USA) were used.

### 2.2. Fungal Isolation and Macro and Micromorphological Identification

Soil samples were collected from two agricultural plots cultivating prickly pear cactus (*Opuntia* spp.) in Colima, Mexico. At each site, three composite soil samples were prepared using the “golden five” sampling method. Each sample comprised 10 subsamples (50 g each), collected at 15 cm from the cactus stem and to a depth of 15 cm. For fungal isolation, each composite sample was homogenized and passed through a 2 mm sieve. A 5 g portion of the homogenized sample was suspended in 50 mL of sterile 0.9% NaCl solution supplemented with 0.01% Tween 80 and then shaken for 30 min at 120 rpm at room temperature. The sample was serially diluted (10^−2^ and 10^−3^), and 100 μL of each dilution was plated onto modified potato dextrose agar (PDAM), which consisted of PDA supplemented with 1% yeast extract (MCD Lab, Mexico) and 250 mg/L chloramphenicol. The plates were incubated for 5–7 days at 25 °C [11]. A sample from each emerging fungal colony was transferred to the center of a fresh Petri dish containing PDA and incubated under identical conditions.

For the macroscopic characterization of isolated fungi, several factors were considered, including the color and surface features of the colonies (which may appear granular, mound-like, floury or slippery), the texture (such as smooth, rough, powdery, or cottony), consistency (either hard or soft), zonation, growth area, as well as the patterns of radial and concentric lines. Additionally, the reverse coloration and surface morphology (flat, convex, or umbonate) were examined [21].

For micromorphological characterization, modified Riddell microcultures were prepared as described by Riddell (1950) [22]. The procedure involved placing moistened filter paper at the bottom of a 9.5 cm Petri dish, then adding 500 μL of sterile distilled water to provide humidity. A sterile glass slide was positioned on the filter paper, and two sections of PDA medium (MCD Lab, Mexico), each approximately 1 cm^2^, were placed onto the slide. Using a sterile bacteriological loop, a fragment of mycelium was inoculated onto each corner of the PDA sections, which were subsequently covered with coverslip. The microcultures were incubated at 25 °C for 7 to 15 days. For further observation, slides were prepared using a lactophenol-cotton blue stain (10:1) and examined under an optical microscope (Motic, Xiamen, China) with 10× and 40× objectives [21].

### 2.3. Taxonomic Identification

The taxonomic identity of the guttation-producing fungi was determined by PCR amplification and Sanger sequencing of the internal transcribed spacer (ITS) region of ribosomal DNA. Additionally, partial sequences of the elongation factor 1-alpha (TEF1-α) and β-tubulin (TUB2) genes were also analyzed. The primers used for these amplifications were ITS5/ITS4 for the ITS region, EF1-728F/EF1-986R for TEF1-α, and Bt2a/Bt2b for TUB2 [23,24,25]. The PCR products were sequenced using an Applied Biosystems 3130 genetic analyzer at the Laboratorio de Diagnóstico Integral Fitosanitario (LADIFIT) in the Estado de Mexico, Mexico. The resulting sequences were compared with reference sequences in GenBank using BLAST analysis (https://blast.ncbi.nlm.nih.gov/Blast.cgi?PROGRAM=blastn&PAGE_TYPE=BlastSearch&LINK_LOC=blasthome, (accessed on 26 January 2026)). The nucleotide sequences reported here have been deposited in GenBank (NCBI) (accession numbers for the ITS region for fungus 1 are PX904639 and PX904640 for fungus 2). The sequences of closely related strains (Table S1) were aligned using the MAFFT v7 program [26]. Multiple alignment of sequences curation was performed with BMGE (Block Mapping and Gathering with Entropy) software v1.12_1 (https://ngphylogeny.fr/tools/tool/273/form, (accessed on 18 February 2026)) [27,28]. Maximum-likelihood (ML) trees were then constructed using IQ-TREE2 v2.4.0 [29]. All phylogenetic trees were created using the ModelFinder function implemented in IQ-TREE2 v2.4.0 (-m MFP), and 10,000 bootstrap replications were performed to assess their robustness. FigTree v1.4.4. (http://tree.bio.ed.ac.uk/, (accessed on 18 February 2026)) was used to visualize and edit phylogenetic trees.

### 2.4. Guttation Collection

Three weeks after reseeding, guttation droplets were collected using a micropipette and transferred into separate sterile 1.5 mL Eppendorf vials, which were then refrigerated at 4 °C. The fluid droplets were filtered through a sterile 13 mm diameter syringe filter fitted with a 0.45 µm polyvinylidene fluoride (PVDF) membrane (Merck, Darmstadt, Germany) to retain the spores [30].

### 2.5. Reactivation Bacterial Strains

The bacterial strains were inoculated into a tube containing Mueller-Hinton broth (MHB), using a sterile inoculation loop to transfer a sample from the blood agar plate. The tube was then incubated at 37 °C for 24 h. Following incubation, the bacterial contents of the MHB tube were transferred to a new tube, and the culture was adjusted to match the 0.5 McFarland turbidity standard. This bacterial suspension was approximately equivalent to 1.5–2.0 × 10^8^ CFU/mL.

### 2.6. Antibacterial Evaluation Using the Agar Well Diffusion Method

The antimicrobial activity of the guttation was evaluated using the well-diffusion method on Mueller-Hinton agar plates. The bacterial panel included in this study was *E. coli* ATCC 25922, *E. coli* ATCC 35218, *S. enterica* serotype Typhimurium ATCC 14028, *S. aureus* ATCC 25923, and *E. faecalis* ATCC 29212, as well as clinical isolates of *K. pneumoniae*, *S. marcescens*, *P. mirabilis*, and *C. freundii*, which were adjusted to a concentration of 1.5–2.0 × 10^8^ CFU/mL. Each bacterial strain was inoculated separately with a swab and allowed to stand for 30 s [31]. Seven wells (6 mm in diameter) were then made in the agar using a sterile hole punch, and the agar plugs were removed to form the wells. Subsequently, 40 µL of the guttation was added to each well [31]. A negative control well containing sterile water was included, and a disc impregnated with 10 µg of gentamicin (BD, USA) served as the positive control. The plates were incubated at 37 °C for 24 h, after which the inhibition zones were measured in millimeters using a vernier caliper [32]. Each assay was performed in triplicate.

### 2.7. Microbroth Dilution Assay for Determination of Minimum Inhibitory Volume (MIV) and Minimum Bactericidal Volume (MBV)

The minimum inhibitory volume (MIV) and minimum bactericidal volume (MBV) of the fungal guttation droplets were determined using a 96-well microbroth dilution assay adapted from CLSI document M07 [33,34]. Assays were performed in sterile, flat-bottom polystyrene microplates (SARSTEDT, Nümbrecht, Germany). Serial dilutions of the fluid droplets were prepared in MHB to obtain tested volumes ranging from 80 to 5 µL per well, and each well was adjusted to a final volume of 200 µL with MHB. Bacterial inoculum was adjusted to a 0.5 McFarland standard (approximately 1–2 × 10^8^ CFU/mL) and subsequently used to inoculate the corresponding wells.

The following controls were included in each assay: (i) growth control (bacterial inoculum + MHB without guttation), (ii) medium sterility control (MHB without inoculum and without guttation), (iii) sterile guttation control (guttation + MHB without inoculum) to rule out intrinsic turbidity of the guttation, and (iv) positive antimicrobial control consisting of gentamicin at 10 µg/mL [35].

### 2.8. Determination of MIV and MBV

Microplates were incubated at 37 °C for 24–48 h under aerobic conditions. The MIV was determined by visual inspection and defined as the lowest guttation volume (µL/well) that inhibited no visible turbidity compared to the growth control. To determine MBV, 100 µL from wells corresponding to guttation volumes equal to or greater than the MIV were -spread-inoculated onto sterile Mueller–Hinton agar (MHA; Bioxon^®^, Kowale, Poland) plates and incubated at 37 °C for 24–48 h under aerobic conditions. MBV was defined as the lowest guttation volume (µL/well) that resulted in no visible bacterial growth on the agar surface. Each determination was performed in duplicate on two different days to ensure reproducibility.

### 2.9. Classification of Bacteriostatic or Bactericidal Effect

The antimicrobial effect of each guttation was categorized using the MBV/MIV ratio. Specifically, the effects were classified as: bactericidal when the MBV/MIV ratio is less than or equal to 4, and bacteriostatic when the ratio is greater than 4. These classifications are based on internationally accepted criteria CLSI susceptibility testing methods (e.g., CLSI M07 and CLSI M26) [33,34,36]. The MBV/MIV ratio was calculated for each guttation–bacterium combination to determine its corresponding biological interpretation.

### 2.10. Statistical Analysis

For the agar well-diffusion assay, inhibition zone diameters were expressed as mean ± standard deviation. Data were analyzed by one-way analysis of variance (ANOVA), and means were compared using Tukey’s test (α = 0.05) in Jamovi v2.6.26 (https://www.jamovi.org/download.html, (accessed on 3 March 2025)).

## 3. Results and Discussions

### 3.1. Morphological Description

Two fungal strains were isolated from microcultures established from the environment surrounding *Opuntia* spp. (prickly pear). Both isolates produced guttation droplets but exhibited distinct macroscopic characteristics. Despite these differences in colony morphology, their microscopic features suggest that both belong to the genus *Penicillium*. Specifically, both isolates present monoverticillate and diverticulate conidiophores bearing hyaline, spherical, smooth-walled conidia formed in chains, characteristic features of the genus [25].

**Figure 1:**
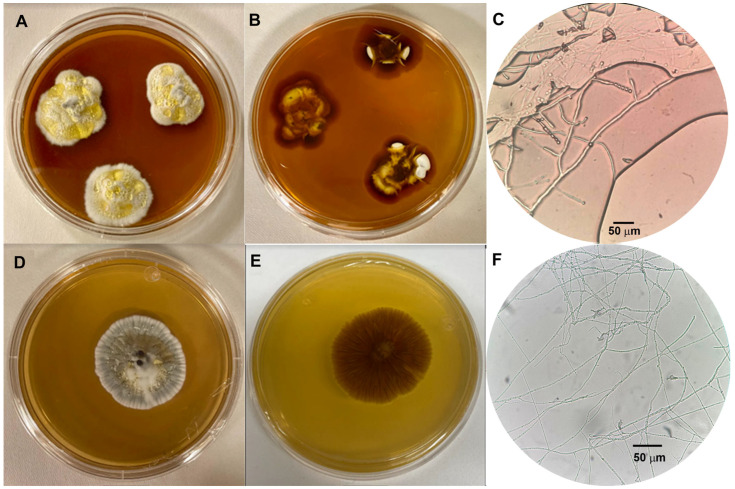
Morphological characterization of guttation-producing fungal isolates. The front (**A**) and reverse (**B**) views show the colonies of fungus 1 after seven days of growth at 25 °C on PDAM, both exhibiting yellow guttation droplets on the colony surface. Similarly, the front (**D**) and reverse (**E**) views show the colonies of fungus 2 under the same conditions, with yellow guttation droplets also observed. In the micrograph of fungus 1 (**C**), structures such as conidiophores and conidia are visible at a magnification of 40×. In contrast, the 10× micrograph of fungus 2 (**F**) reveals tubular hyphae, conidiophores, metulae, and phialides.

Fungus 1 formed irregular colonies with rapid vegetative growth. On the obverse side, the colony appeared white, with a velvety texture, was slightly elevated in some areas, and displayed a wavy margin (Figure 1A). A distinctive feature was the presence of yellow guttation droplets. The reverse side of the colony maintained the wavy margin and showed compact, irregular growth (Figure 1B). The central region was characterized by dark brown pigmentation surrounding an irregular, whitish area.

In contrast, fungus 2 developed a rapidly growing, white, filamentous colony. The colony exhibited a velvety texture with localized raised areas, and a threadlike margin, and produced yellow guttation droplets (Figure 1C). On the reverse side, the colony featured a threadlike margin and light brown pigmentation in the center, which gradually transitioned to light yellow towards the periphery. Notably, striations extended radially outward from the central region (Figure 1D).

### 3.2. Taxonomic Identification

**Figure 2:**
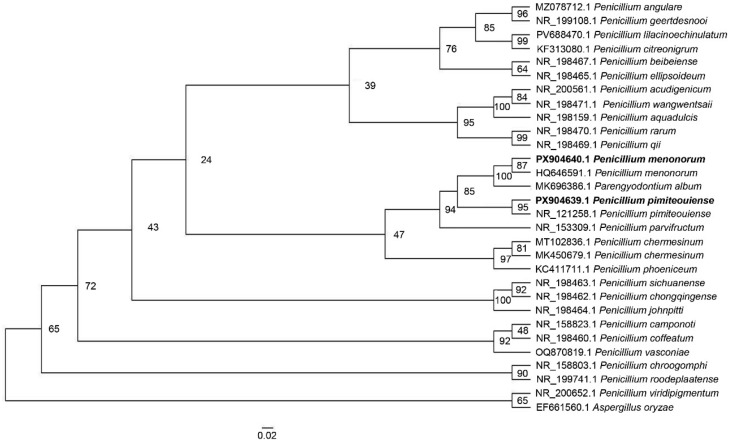
Phylogenetic analysis of the fungal 18S-ITS-5.8S-ITS2-28S ribosomal DNA regions from *P. pimiteouiense* and *P. menonorum* obtained from soil samples in two prickly pear cactus (*Opuntia* spp.) agricultural plots. Thirty sequences were considered for analysis; those highlighted in bold were included in this study. The best-fit model, TIM2e + I + R2, was determined using IQ-TREE2. Bootstrap values (10,000 replicates) are displayed at each branch as a percentage. The scale bar indicates substitutions per site.

A BLAST search revealed that the ITS and β-tubulin sequences of fungus 1 were 100% identical to those of *Penicillium pimiteouiense* (GenBank NR_121258.1 and HQ646567.1, respectively). In contrast, the TEF1-α sequence showed 91.19% identity with a *Penicillium* sp. (GenBank OR795058.1). For fungus 2, the ITS and β-tubulin sequences showed 100% and 99.77% identity, respectively, with sequences from *Penicillium menonorum* (GenBank HQ646591.1 and OP562832.1, respectively). However, the TEF1-α sequence of fungus 2 was 90.45% identical to that of a *Penicillium* sp. (GenBank OR795058.1). Phylogenetic analysis of the ITS sequences was carried out by tree construction. The ITS sequences of fungus 1 (*P. pimiteouiense*, GenBank PX904639.1) and fungus 2 (*P. menonorum*, GenBank PX904640.1) were aligned with available strain sequences of *P. pimiteouiense* and *P. menonorum*, their nearest species (Table S1). The phylogenetic analysis revealed that these fungi were grouped with reference isolates of *P. pimiteouiense* and *P. menonorum*, with bootstrap support of 95 and 87%, respectively (Figure 2). These were different from other *Penicillium* spp., which were grouped in distinct clades. These findings suggest that the isolated fungi are closely related to *P. pimiteouiense* and *P. menonorum*. Based on morphological properties, sequence homology, and phylogenetic analysis, fungus 1 and fungus 2 were identified as *P. pimiteouiense* and *P. menonorum*, respectively.

### 3.3. Antibacterial Activity

Guttation droplets obtained from *P. pimiteouiense* and *P. menonorum* exhibited measurable antibacterial activity in the agar well diffusion assay. No inhibition halo was observed for the negative control (sterile water), confirming the absence of plate-related artifacts. Overall, the inhibition profiles differed between bacterial species and fungal sources.

#### 3.3.1. Agar Well Diffusion

The fungal guttations of *P. pimiteouiense* and *P. menonorum* produced measurable inhibition zones against several bacteria (Table 1). Overall, *P. pimiteouiense* generated larger inhibition zones than *P. menonorum* for most Gram-negative organisms, including *E. coli* ATCC 25922 (22.7 ± 0.9 mm vs. 14.4 ± 0.8 mm), *S. enterica* serovar Typhimurium ATCC 14028 (24.4 ± 0.2 mm vs. 16.5 ± 0.3 mm), *K. pneumoniae* (clinical isolate) (21.4 ± 0.1 mm vs. 19.2 ± 0.2 mm), *S. marcescens* (clinical isolate) (21.3 ± 0.6 mm vs. 16.1 ± 0.3 mm), and *C. freundii* (clinical isolate) (15.2 ± 1.0 mm vs. 13.4 ± 0.8 mm). For *S. aureus* ATCC 25923, both exudates showed similar inhibition diameters (18.6 ± 0.6 mm and 19.8 ± 1.0 mm). Gentamicin (10 µg) produced inhibition zones within the expected range for the evaluated bacteria (Table 1). For statistical inference, inhibition zone diameters were analyzed separately for each bacterium using one-way ANOVA followed by Tukey’s HSD test (α = 0.05). Across the Gram-negative panel, gutattion 1 was significantly higher than guttation 2, and in several cases its activity was statistically comparable to gentamicin (Table 1). In contrast, for *S. aureus* both guttations were statistically indistinguishable from each other but significantly lower than gentamicin. For *S. enterica* serovar Typhimurium, all treatments differed significantly, with guttation 1 showing the greatest inhibition, followed by gentamicin and guttation 2. Statistically significant pairwise differences are indicated by different letter groupings in Table 1, whereas treatments sharing the same letter are not significantly different.

Research on the antimicrobial activity of fungal guttation droplets from the genus *Penicillium* against medically important bacteria remains scarce. In one of the few available studies, Gunawardena et al. (2025) [7] reported inhibition zones of 12 mm and 6 mm for *S. aureus* and *E*. *coli*, respectively, using exudates from *Penicillium* strains. These values are considerably lower than those obtained in the present study, where exudates from *P*. *pimiteouiense* and *P. menonorum* produced inhibition zones of 22.7 mm and 14.4 mm against *E. coli*, and 18.6 mm and 19.8 mm against *S. aureus*. The differences in bacterial susceptibility could be attributed to the presence of specific secondary metabolites, which may include various phenolic compounds and other complex polycyclic aromatic structures. These compounds have been extensively documented in filamentous fungi and, in many cases, have been associated with relevant biological activities.

The antimicrobial activity of the genus *Penicillium* is well documented, particularly with organic solvent extracts. For instance, Zerroug et al. (2018) reported inhibition zones of 37.5 mm for *S. aureus* and 45.5 mm for *E. coli* using ethyl acetate extracts of *Penicillium griseofulvum* [37]. These values are approximately double those observed in our study with exudates. The higher inhibitory activity of organic extracts compared to guttation droplets could be due to multiple factors, including fungal species, culture conditions, extraction method, and bioactive compounds concentration. The extraction process with solvents such as ethyl acetate, followed by evaporation, enables the concentration of secondary metabolites from a large volume of culture broth. In contrast, the guttation droplets were evaluated directly, without any concentration step, thus reflecting the basal concentration at which the fungus excretes these compounds [7].

However, Basavarajappa et al. (2023) reported inhibition zones of 21.6 mm and 19.48 mm for *E. coli* and *S. aureus*, respectively, using ethyl acetate extracts of *Penicillium limosum* [38]. These values are close to those obtained with the exudates of *P. pimiteouiense* and *P. menonorum* in the present study. Similarly, Chemmam et al. (2024) reported inhibition zones of 17.2 mm for *E. coli*, 18.2 mm for *K. pneumoniae*, 11.1 mm for *S. typhimurium*, and 25.3 mm for *S. aureus* using ethyl acetate extracts of *Penicillium biliae* [39]. These results are also comparable to those obtained with our exudates, suggesting that the latter possess antimicrobial potential similar to that of extracts obtained with organic solvents. This similarity is particularly relevant, as obtaining fungal exudates is a more direct method and avoids the use of organic solvents, which could have positive implications for both economic and environmental perspectives.

#### 3.3.2. Microbroth Dilution Assay (MIV/MBV)

Minimum inhibitory volumes (MIV) ranged from 10 to >80 µL/well (5 to >40% *v*/*v*), depending on the bacterium and exudate (Table 2). The lowest MIV was observed for *C. freundii* with *P. pimiteouiense* (10 µL/well; 5% *v*/*v*). *P. menonorum* showed low MIV values against *E. coli* ATCC 25922 and *S. marcescens* (20 µL/well; 10% *v*/*v*). Minimum bactericidal volumes (MBV) reached within the tested range (≤80 µL/well) were observed for selected combinations (Table 3), including *P. pimiteouiense* against *E. coli* ATCC 35218 (80 µL/well) and *S. aureus* ATCC 25923 (80 µL/well), and *P. menonorum* against *S. enterica* serovar Typhimurium ATCC 14028 (80 µL/well), *K. pneumoniae* (60 µL/well), and *C. freundii* (40 µL/well). For several exudate–bacterium combinations, a bactericidal activity was not observed within the tested range (MBV > 80 µL/well), indicating limited bactericidal efficacy under the evaluated conditions.

These results indicate that fungal guttation droplets exert both inhibitory and lethal effects on bacteria, depending on the specific bacterial target and the fungal species involved. However, since the tested material was crude guttation droplets rather than a purified compound, the MIV and MBV values serve as comparative parameters in this study. They are not directly equivalent to MIC/MBC values (expressed in µg/mL) reported for purified antimicrobials [40]. This distinction is important because broth microdilution, considered the most quantitative conventional method for determining inhibitory potency, depends on adequate inoculum standardization and on the absence of visual interference caused by turbidity, precipitation, or coloration of the tested sample [41,42].

A particularly noteworthy finding was the inconsistent behavior observed in *P. mirabilis*. In the agar well diffusion assay, both guttation droplets produced moderate inhibition halos, measuring approximately 16.5–16.7 mm, suggesting measurable antibacterial activity. However, this pattern did not consistently replicate in the microbroth dilution assay, where inhibitory and bactericidal endpoints showed lower consistency across the tested range. This discrepancy may reflect methodological sensitivity rather than a true contradiction in antibacterial potency.

One plausible explanation concerns the characteristic swarming motility of *P. mirabilis* on solid surfaces. Sub-inhibitory concentrations of antimicrobials can suppress swarming and swimming motility in *P. mirabilis* without noticeably impairing planktonic growth. A recent in vitro study showed that sub-MIC ciprofloxacin significantly inhibited the swarming and swimming abilities of *P. mirabilis* without noticeably affecting its growth. This finding highlights that behavioral changes associated with surfaces can occur independently of the inhibition observed in broth culture. Additionally, if components of the guttation droplets interfered with swarming or surface expansion, they could generate visible halos in agar without necessarily producing low MIV or MBV values in liquid medium [43]. For this reason, the *P. mirabilis* discrepancy should be discussed as likely due to a combination of biological behavior and assay-specific limitations rather than as evidence against antibacterial activity.

#### 3.3.3. Bacteriostatic vs. Bactericidal Classification

Based on the MBV/MIV ratio criterion (≤4 bactericidal; >4 bacteriostatic), bactericidal behavior was confirmed for *P. pimiteouiense* against *E. coli* ATCC 35218, *K. pneumoniae*, and *S. aureus*, and for *P. menonorum* against *S. enterica* serovar Typhimurium, *K. pneumoniae*, and *C. freundii*. Bacteriostatic behavior was observed for *P. pimiteouiense* against *C. freundii* (MBV/MIV = 8) and for *P. menonorum* in cases where MBV exceeded the tested range while MIV was low (e.g., MIV = 20 µL/well; Table 4). For combinations with MBV > 80 µL/well and MIV ≥ 40 µL/well, classification should be interpreted as indeterminate within the tested range.

Our results suggest that fungal guttation droplets from *P. pimiteouiense* and *P. menonorum* constitute a distinct, underexplored alternative for bioprospecting, offering a strategic pathway for identifying new classes of antibacterial agents. While the antibacterial activity demonstrated here is robust, we acknowledge that the chemical composition of guttation droplets is dynamic and may vary with the fungal growth phase and nutrient availability. Although preliminary internal validations showed consistent bioactivity across different collection times, the lack of high-resolution chemical characterization, such as LC-MS/MS, remains a limitation. Future studies focusing on the metabolomic profiling of these guttations will be essential to identify a specific bioactive compound and fully elucidate their chemical stability.

## 4. Conclusions

This study demonstrates that guttation droplets produced by *P. pimiteouiense* and *P. menonorum* constitute a distinct, poorly investigated alternative for bioprospecting, offering a strategic pathway for identifying new classes of antibacterial agents. Our results, derived from standardized agar well diffusion and microbroth dilution assays, consistently showed that these guttation droplets inhibit both Gram-negative and Gram-positive bacteria, including *E. coli*, *S. enterica*, *K. pneumoniae*, and *S. aureus*. Notably, the inhibition zones produced by *P. pimiteouiense* were comparable to those of the antibiotic gentamicin, underscoring the potency of these natural secretions. Quantitative evaluation by microbroth dilution revealed minimum inhibitory volumes ranging from 10 to >80 µL/well, while MBV/MIV ratios confirmed bactericidal nature of specific pathogen–guttation combinations. To address the inherent complexity of fungal droplets, the reproducibility of these antimicrobial effects was observed across multiple assays, highlighting fungal guttation as a reliable reservoir of bioactive metabolites. Unlike conventional solvent-based extraction, direct evaluation of guttation droplets captures the natural secretion of secondary metabolites, providing a rapid, environmentally friendly approach for screening.

While these findings validate the biological potential of *Penicillium* guttation, further studies are warranted to fully characterize its chemical profile using metabolomic and spectrometric approaches (LC-MS/MS). Elucidating the specific mechanisms of action will be crucial to translating these preliminary findings into therapeutic candidates. Overall, this work supports fungal guttation as a promising, underexplored strategy for discovering novel antimicrobial compounds, contributing to the global effort to combat antimicrobial resistance.

## Figures and Tables

**Table 1:** Inhibition zones produced by guttation droplets from *Penicillium pimiteouiense* and *Penicillium menonorum* against clinically relevant bacterial strains in the agar well diffusion assay.

Strains	*P. pimiteouiense* (mm)	*P. menonorum* (mm)	Gentamicin (mm)
*E. coli* (ATCC 25922)	22.7 ± 1.0 ^a^	14.4 ± 0.8 ^b^	21.7 ± 0.5 ^a^
*E. coli* (ATCC 35218)	19.2 ± 0.5	13.5 ± 0.5	22.1 ± 0.6
*S.* Typhimurium (ATCC 14028)	24.4 ± 0.2 ^a^	16.5 ± 0.3 ^c^	23.1 ± 0.3 ^b^
*K. pneumoniae* (clinical isolate LRS)	21.4 ± 0.1 ^a^	19.2 ± 0.1 ^b^	21.9 ± 0.5 ^a^
*S. marcescens* (clinical isolate HVR)	21.3 ± 0.1 ^a^	16.1 ± 0.3 ^b^	20.9 ± 0.5 ^a^
*P. mirabilis* (clinical isolate MCV)	16.7 ± 0.5 ^a^	16.5 ± 1.2 ^a^	17.9 ± 0.2 ^a^
*S. aureus* (ATCC 25923)	18.6 ± 0.6 ^b^	19.8 ± 1.0 ^b^	21.5 ± 0.3 ^a^
*E. faecalis* (ATCC 29212)	15.2 ± 0.4	11.4 ± 0.7	18.8 ± 0.4
*C. freundii* (clinical isolate GAS)	15.2 ± 1.0 ^b^	13.4 ± 0.8 ^b^	26.4 ± 0.5 ^a^

Note: Values are expressed as mean ± standard deviation of inhibition zone diameter (mm). Gentamicin (10 µg) was used as the positive control. ^a, b, c^ indicate significant differences among treatments (one-way ANOVA, followed by Tukey’s HSD, α = 0.05; *n* = 3).

**Table 2:** Minimum inhibitory volume (MIV) of guttation droplets from *Penicillium pimiteouiense* and *Penicillium menonorum* against clinically relevant bacterial strains.

Strains	Fungal Guttation Droplets			
	*P. pimiteouiense*		*P. menonorum*	
	MIV (µL/well)	MIV (% *v*/*v*)	MIV (µL/well)	MIV (% *v*/*v*)
*E. coli* (ATCC 25922)	40	20	20	10
*E. coli* (ATCC 35218)	60	30	60	30
*S.* Typhimurium (ATCC 14028)	40	20	60	30
*K. pneumoniae* (clinical isolate LRS)	40	20	40	20
*S. marcescens* (clinical isolate HVR)	80	40	20	10
*P. mirabilis* (clinical isolate MCV)	>80	-	80	40
*S. aureus* (ATCC 25923)	20	10	40	20
*E. faecalis* (ATCC 29212)	80	40	80	40
*C. freundii* (clinical isolate GAS)	10	5	20	10

Note: MIV was determined by microbroth dilution and defined as the lowest guttation volume (µL/well) that showed no visible turbidity relative to growth control. Values are reported as µL/well and as a percentage of the final well volume (% *v*/*v*). A hyphen indicates that the MIV percentage was not determined.

**Table 3:** Minimum bactericidal volume (MBV) of guttation droplets from *Penicillium pimiteouiense* and *Penicillium menonorum* against clinically relevant bacterial strains.

Strains	Fungal Guttation Droplets			
	*P. pimiteouiense*		*P. menonorum*	
	MBV (µL/well)	MBV (% *v*/*v*)	MBV (µL/well)	MBV (% *v*/*v*)
*E. coli* (ATCC 25922)	>80	-	>80	-
*E. coli* (ATCC 35218)	80	40	>80	-
*S.* Typhimurium (ATCC 14028)	>80	-	80	40
*K. pneumoniae* (clinical isolate LRS)	80	40	60	30
*S. marcescens* (clinical isolate HVR)	>80	-	>80	-
*P. mirabilis* (clinical isolate MCV)	>80	-	>80	-
*S. aureus* (ATCC 25923)	80	40	>80	-
*E. faecalis* (ATCC 29212)	>80	-	>80	-
*C. freundii* (clinical isolate GAS)	80	40	40	20

Note: MBV was determined as the lowest guttation volume (µL/well), resulting in no visible bacterial growth after subculture onto Mueller–Hinton agar. Values are reported as µL/well and as a percentage of the final well volume (% *v*/*v*). A hyphen indicates that the bactericidal endpoint was not reached within the tested range.

**Table 4:** Classification of the antimicrobial effect of guttation droplets from *Penicillium pimiteouiense* and *Penicillium menonorum* against clinically relevant bacterial strains based on the MBV/MIV ratio.

Strain	Fungal Guttation Droplets	MBV (µL/well)	MIV (µL/well)	MBV/MIV	Classification
*E. coli* 25922	1	40	>80	-	Bacteriostatic
	2	20	>80	-	Bacteriostatic
*E. coli* 35218	1	60	80	1.33	Bactericidal
	2	60	>80	-	Bacteriostatic
*S.* Typhimurium 14028	1	40	>80	-	Bacteriostatic
	2	60	80	1.33	Bactericidal
*K. pneumoniae* WT	1	40	80	2	Bactericidal
	2	40	60	1.5	Bactericidal
*S. marcescens* WT	1	80	>80	-	Bacteriostatic
	2	20	>80	-	Bacteriostatic
*P. mirabilis*	1	>80	>80	-	Bacteriostatic
	2	80	>80	-	Bacteriostatic
*S. aureus* 25923	1	20	80	4	Bactericidal
	2	40	>80	-	Bacteriostatic
*E. faecalis* 29212	1, 2	80	>80	-	Bacteriostatic
*C. freundii*	1	10	80	8	Bacteriostatic
	2	20	40	2	Bactericidal

Note: Antimicrobial activity was classified as bactericidal when the MBV/MIV ratio was ≤4 and bacteriostatic when the ratio was >4. In cases where the MBV > 80 µL/well and the MIV falls within the tested range, the bactericidal endpoint was not reached under the evaluated conditions. A hyphen indicates that the MBV/MIV ratio was not determined.

## Data Availability

The original contributions presented in this study are included in the article/Supplementary Materials. Further inquiries can be directed to the corresponding author.

## Supplementary Materials

The following supporting information can be downloaded at: https://www.mdpi.com/article/10.3390/jof12040262/s1, Table S1. Strains used in phylogenetic analysis of the 18S-ITS-5.8S-ITS2-28S ribosomal DNA regions.
