# Supplementary material for: Antibacterial Activity of Guttation Droplets from Penicillium pimiteouiense and Penicillium menonorum Against Clinically Relevant Bacterial Pathogens
Source: J Fungi (Basel). 2026 Apr 3;12(4):262. doi: 10.3390/jof12040262 (PMC13117783; doi:10.3390/jof12040262)
Supplement: Supplementary file 1 [file jof-12-00262-s001.zip › jof-4220728-supplementary.pdf]

Journal of Fungi

MDPI

Table S1. Strains used in phylogenetic analysis of the 18S-ITS-5.8S-ITS2-28S ribosomal DNA regions.

Access number GenBank	Species name
PX904640.1	*Penicillium menonorum*
HQ646591.1	*Penicillium menonorum*
PX904639.1	*Penicillium pimiteouiense*
NR_121258.1	*Penicillium pimiteouiense*
MT102836.1	*Penicillium chermesinum*
OQ870819.1	*Penicillium vasconiae*
MZ078712.1	*Penicillium angulare*
PV688470.1	*Penicillium lilacinoechinulatum*
KF313080.1	*Penicillium citreonigrum*
MK696386.1	*Parengyodontium album*
NR_200652.1	*Penicillium viridipigmentum*
NR_200561.1	*Penicillium acidogenicum*
NR_158823.1	*Penicillium camponoti*
NR_158803.1	*Penicillium chroogomphi*
NR_153309.1	*Penicillium parvifructum*
NR_199741.1	*Penicillium roodeplaatense*
NR_199108.1	*Penicillium geertdesnooi*
NR_198471.1	*Penicillium wangwentsaii*
NR_198470.1	*Penicillium rarum*
NR_198469.1	*Penicillium qii*
NR_198467.1	*Penicillium beibeiense*
NR_198465.1	*Penicillium ellipsoideum*
NR_198464.1	*Penicillium johnpittii*
NR_198463.1	*Penicillium sichuanense*
NR_198462.1	*Penicillium chongqingense*
NR_198460.1	*Penicillium coffeatum*
NR_198159.1	*Penicillium aquadulcis*
KC411711.1	*Penicillium phoeniceum*
MK450679.1	*Penicillium chermesinum*
EF661560.1	*Aspergillus oryzae*

Disclaimer/Publisher's Note: The statements, opinions and data contained in all publications are solely those of the individual author(s) and contributor(s) and not of MDPI and/or the editor(s). MDPI and/or the editor(s) disclaim responsibility for any injury to people or property resulting from any ideas, methods, instructions or products referred to in the content.
